# Chromosome Y Regulates Survival Following Murine Coxsackievirus B3 Infection

**DOI:** 10.1534/g3.111.001610

**Published:** 2012-01-01

**Authors:** Laure K. Case, Leon Toussaint, Mohamad Moussawi, Brian Roberts, Naresha Saligrama, Laurent Brossay, Sally A. Huber, Cory Teuscher

**Affiliations:** * Department of Medicine and; † Department of Pathology, University of Vermont, Burlington, Vermont 05405; ‡ Department of Molecular Microbiology and Immunology and; § Graduate Program in Pathobiology, Division of Biology and Medicine, Brown University, Providence, Rhode Island 02912; ** Center for Nuclear Receptors and Cell Signaling, University of Houston, Houston, Texas 77204

**Keywords:** heart disease, sexual dimorphism, heterochromatin

## Abstract

Coxsackievirus B3 (CVB3) contributes to the development of myocarditis, an inflammatory heart disease that predominates in males, and infection is a cause of unexpected death in young individuals. Although gonadal hormones contribute significantly to sex differences, sex chromosomes may also influence disease. Increasing evidence indicates that Chromosome Y (ChrY) genetic variants can impact biological functions unrelated to sexual differentiation. Using C57BL/6J (B6)-ChrY consomic mice, we show that genetic variation in ChrY has a direct effect on the survival of CVB3-infected animals. This effect is not due to potential *Sry*-mediated differences in prenatal testosterone exposure or to differences in adult testosterone levels. Furthermore, we show that ChrY polymorphism influences the percentage of natural killer T cells in B6-ChrY consomic strains but does not underlie CVB3-induced mortality. These data underscore the importance of investigating not only the hormonal regulation but also ChrY genetic regulation of cardiovascular disease and other male-dominant, sexually dimorphic diseases and phenotypes.

Coxsackievirus infection of the heart can lead to myocarditis and cause unexpected death in individuals less than 40 years old (Woodruff 1980). Like most heart diseases, myocarditis predominates in males by a 2:1 ratio and displays a greater severity of disease over females. Susceptibility is greatest in men during the first year of life and after puberty. However, females are often rendered susceptible to myocarditis during pregnancy, during the postpartum period, and in individuals over 40 years of age, suggesting a role for hormonal influences on disease development (Woodruff 1980).

The coxsackievirus B3 (CVB3)-induced mouse model of experimental myocarditis exhibits many of the same characteristics as the human disease (Gauntt *et al.* 1979; Grodums and Dempster 1959b; Huber *et al.* 1982; Matsumori and Kawai 1980; Reyes and Lerner 1985; Wong *et al.* 1977). Adult male mice less than 40 weeks of age are more susceptible to CVB3-induced myocarditis than females of the same age. However, both males and females less than 3 weeks of age or over 40 weeks of age are equally susceptible, indicating that age-related changes in sex-associated hormones may influence experimental myocarditis susceptibility (Grodums and Dempster 1959a; Lyden *et al.* 1987a). In this regard, susceptibility was significantly reduced in male mice that were orchiectomized prior to infection (Huber *et al.* 1982). However, when orchiectomized males received exogenous testosterone or progesterone prior to infection, disease susceptibility returned to similar levels seen in intact males (Huber *et al.* 1982). In contrast, treatment of orchiectomized males with estrogens provided protection from disease. Moreover, *in vitro* experiments revealed that male and female cardiomyocytes exhibited enhanced infectivity when cultured in the presence of progesterone or testosterone, but not estradiol, due to increased virus binding to the cell surface (Lyden *et al.* 1987b). Further studies demonstrated that testosterone induced an approximately 6-fold increase in virus receptor expression in cardiomyocytes (Lyden *et al.* 1987a). Therefore, these studies indicate that androgens enhance virus receptor expression and disease susceptibility, whereas estrogens provide protection from disease.

Sex hormones also influence the immune response mounted against CVB3 infection in mice. Susceptibility to CVB3-induced myocarditis in males is dependent on the induction of a CD4^+^ Th1 cellular response, and the immune bias toward a Th1 phenotype requires activated γδ Tcells (Huber and Pfaeffle 1994; Huber *et al.* 2001). Orchiectomy led to an increase in immune regulatory cells in the heart, including regulatory T cells, suggesting that testosterone inhibits anti-inflammatory cell recruitment and enhances susceptibility to disease (Frisancho-Kiss *et al.* 2009). In contrast, resistance to disease in females was associated with a preferential Th2 response to infection (Huber and Pfaeffle 1994). However, treatment of female mice with testosterone *in vivo* caused a shift from the protective Th2 cell response toward the Th1 phenotype, leading to enhanced susceptibility in these mice (Huber and Pfaeffle 1994).

Clearly, testosterone plays a critical role in the sexual dimorphism in susceptibility to myocarditis by regulating not only viral infectivity but also differences in innate and adaptive antiviral immune responses. While a clear role for sex-associated hormones, and particularly testosterone, can be observed in the male-specific sexual dimorphism during CVB3 pathogenesis, the effect of Chromosome Y (ChrY) on disease has not been examined. Here, we provide direct evidence that ChrY regulates survival following CVB3 infection and suggest that the mechanism is unrelated to biological differences in testosterone production among the strains.

## Materials and Methods

### Mice

C57BL/6J, C57BL/6J-ChrY^129S1/SvImJ^/NaJ, C57BL/6J-ChrY^A/J^/NaJ, C57BL/6J-ChrY^MET^/J, C57BL/6JEi-ChrY^AKR/J^/EiJ, C57BL/6JEi-ChrY^BUB/BnJ^/EiJ, C57BL/6JEi-ChrY^LEWES^/EiJ, C57BL/6JEi-ChrY^RF/J^/EiJ, C57BL/6JEi-ChrY^SJL/J^/EiJ, C57BL/6JEi-ChrY^ST/bJ^/EiJ, C57BL/6JEi-ChrY^WSB/Ei^/EiJ, B6Ei.MA-*A*ChrY^MA/MyJ^/EiJ, and B6Ei.SWR-*A*ChrY^SWR/J^/EiJ were purchased from the Jackson Laboratory (Bar Harbor, ME) and bred and maintained on the C57BL/6J background in the animal facility at the University of Vermont. B6-ChrY^129F11/Pas^ and B6-ChrY^Nkt-129/Pas^ mice were obtained from the Pasteur Institute (Paris, France) and bred and maintained in the animal facility at Brown University. Only male mice were used in the experiments. All animal protocols were approved by the Animal Care and Use Committees of the University of Vermont and Brown University.

### Virus and infection

The H3 variant of CVB3 was made from an infectious cDNA clone as previously described (Knowlton *et al.* 1996). Mice were infected by intraperitoneal injection of 0.5 ml of PBS containing 100 or 50 PFU CVB3 and killed seven days after infection.

### Serum testosterone

Eight-week-old mice were bled by tail vein, and the samples were spun down for 20 min at 16,000 *g* to extract serum. Serum testosterone levels were measured using a testosterone enzyme immunoassay (EIA) kit according to the manufacturer’s instructions (Assay Designs, Ann Arbor, MI). Briefly, serum samples were diluted with 1 part steroid displacement reagent for every 99 parts sample. Each sample was diluted 1:10 and 1:20 in PBS and added to the 96-well plate in duplicate, followed by the primary antibody, and incubated at room temperature for 1 hr while shaking. Wells were washed and conjugate was added to each well and incubated at room temperature for 1 hr while shaking. Wells were washed, and the pNpp substrate was added to each well and incubated at 37° for 1 hr without shaking. Stop solution was added, and the plate was read immediately with optical density at 405 nm.

### Lymphocyte isolation

Perfused and intact liver, spleen, and thymus were extracted from 8-week-old mice and placed in 50 ml tubes containing RPMI 1640 media with 10% FCS and shipped overnight on ice to Brown University. Thymocytes were minced and washed once in 1% PBS-serum. To obtain splenic lymphocytes, spleens were minced, passed through nylon mesh (Tetko, Kansas City, MO), washed once in 1% PBS-serum, and then cell suspensions were layered on lympholyte-M (Cedarlane Laboratories Ltd., Canada). Hepatic lymphocytes were prepared by mincing and passing through a 70 mm nylon cell strainer (Falcon, Franklin Lakes, NJ). After washing three times in 1% PBS-serum, cell suspensions were layered on a two-step discontinuous Percoll gradient (Pharmacia Fine Chemicals, Piscataway, NJ). Splenocytes and hepatic lymphocytes were collected after centrifugation for 20 min at 900 *g*.

### Antibodies and flow cytometric analysis

TCRβ-FITC (or APC), CD8α-efluor 450 (or Pacific blue), CD4-APC (or perCP), CD44-efluor 780, NK1.1-PE-Cy7, and CD24-FITC were purchased from eBioscience (San Diego, CA). CD1d Tetramer-PE was obtained from the National Institute of Allergy and Infectious Disease MHC Tetramer Core Facility at Emory University (Atlanta, GA). Cells were suspended in buffer composed of PBS containing 1% FCS. Cells were first incubated with 2.4G2 mAb and stained with mAbs specific for cell surface markers for 20 min at room temperature. Depending on the experiment and the tissue, from 2.5 × 10^5^ to 1 × 10^6^ events were collected in the lymphoid gate on a FACSAria. The data were analyzed using FlowJo (Tree Star Inc., Ashland, OR).

### Statistics

Statistical analyses were performed using GraphPad Prism version 5.0c (GraphPad Software, San Diego, CA). The specific tests used are detailed in the figure legends. A *P* value ≤ 0.05 was considered significant.

## Results and Discussion

### ChrY polymorphism regulates CVB3-induced mortality

To test whether ChrY polymorphism influences survival following CVB3 infection, we utilized a panel of consomic strains in which C57BL/6J (B6) mice inherit ChrY donated from either *Mus musculus musculus* (*musculus*) or *Mus musculus domesticus* (*domesticus*) subspecies (B6-ChrY). The B6-ChrY consomic strains used include B6-ChrY^SJL^, B6-ChrY^SWR^, B6-ChrY^AKR^, B6-ChrY^MA^, B6-ChrY^ST^, B6-ChrY^LEWES^, B6-ChrY^BUB^, B6-ChrY^RF^, B6-ChrY^MET^, B6-ChrY^WSB^, B6-ChrY^A/J^, and B6-ChrY^129S1^, in which the mouse strain donating ChrY to B6 is indicated in superscript. Male mice from the panel of B6-ChrY consomic strains, as well as wild-type B6 male mice, were infected intraperitoneally with either 100 or 50 PFU CVB3 and monitored for survival for eight days following infection. The survival data obtained from each B6-ChrY consomic strain at each virus dose was compared with wild-type B6 and tested for significant differences using a log-rank (Mantel-Cox) test (supporting information, File S1).

**Figure 1:**
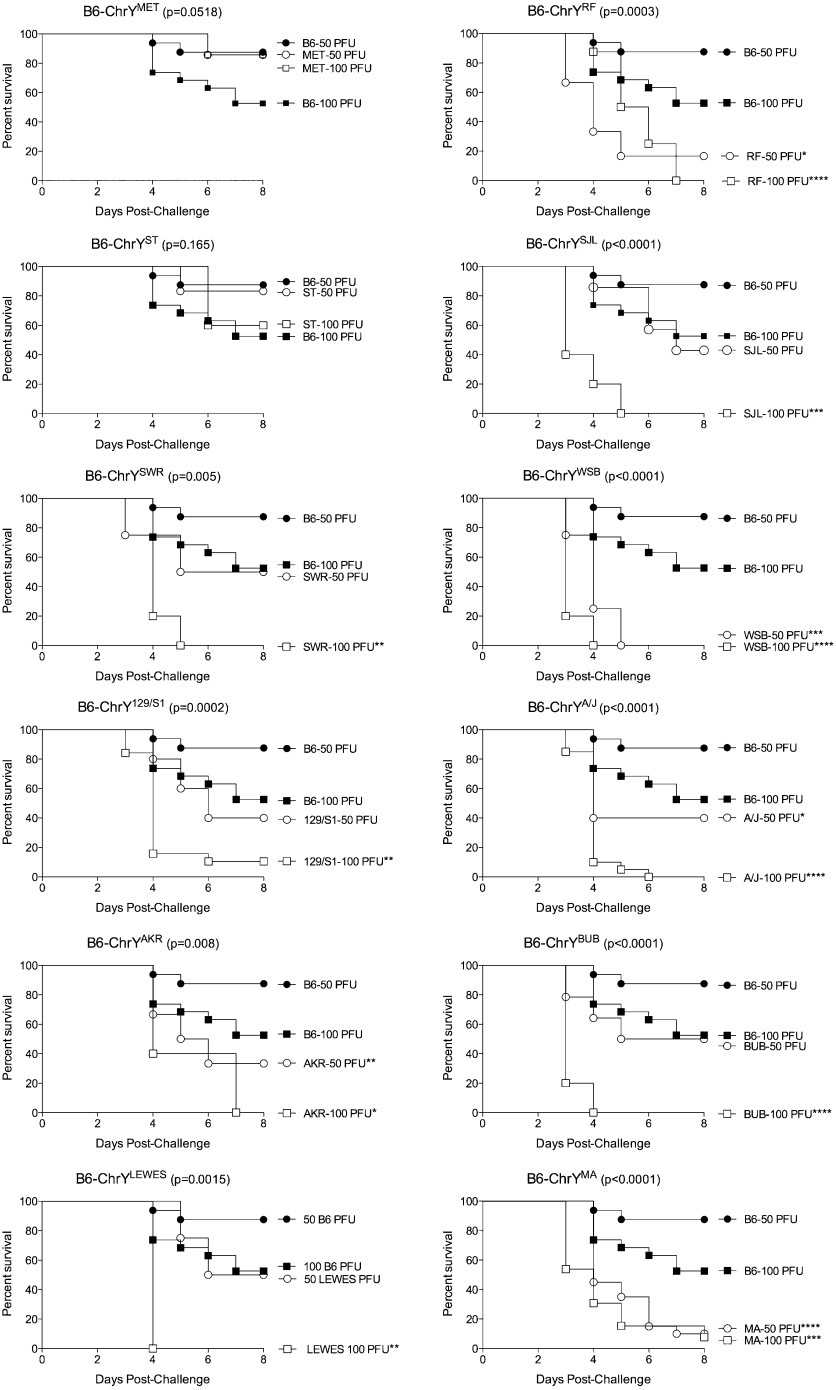
ChrY polymorphism influences susceptibility to CVB3-induced mortality. B6 and B6-ChrY consomic strains were infected with 100 or 50 PFU CVB3 and monitored for survival. Significance of observed differences were determined using a log-rank (Mantel-Cox) test (n = 5–20). The legend labels indicate the mouse strain donating ChrY to B6. *P* values in graph title represent overall *P* values, and asterisks in the legend labels represent significant differences in survival between B6 and the B6-ChrY consomic strains at the indicated virus dose. ^*^P < 0.05; ^**^P < 0.01;^ ***^P < 0.001; ^****^P < 0.0001.

**Figure 2:**
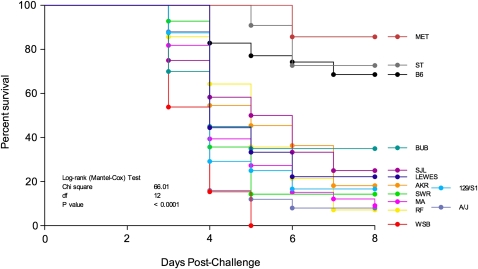
CVB3-induced mortality exhibits a continuous distribution across the B6-ChrY consomic strains. The survival data obtained from the infection with 100 and 50 PFU in Figure 1 was combined for each consomic strain and plotted in a single graph. The legend labels indicate the mouse strain donating ChrY to B6. Significance of observed differences measured using a log-rank (Mantel-Cox) test (n = 5–20).

B6-ChrY^ST^ and B6-ChrY^MET^ mice were the only strains that did not display a decrease in survival following infection with either 100 or 50 PFU CVB3 when compared to B6. B6-ChrY^LEWES^, B6-ChrY^SWR^, B6-ChrY^SJL^, B6-ChrY^129/S1^ and B6-ChrY^BUB^ mice only showed a decrease in survival compared with B6 when infected with 100 PFU CVB3 but not at the 50 PFU dose. Conversely, B6-ChrY^RF^, B6-ChrY^MA^, B6-ChrY^AKR^, B6-ChrY^SWR^, B6-ChrY^WSB^, and B6-ChrY^A/J^ mice were significantly more susceptible to CVB3-induced mortality at both the 100 and 50 PFU doses compared with B6 (Figure 1). Transforming the data into a single susceptibility value by averaging the 100 and 50 PFU response groups for each strain indicated that B6-ChrY consomic strains exhibit a continuous distribution of CVB3-induced mortality phenotypes (Figure 2). Because the only genetic difference among these strains is the inheritance of ChrY, the differences in mortality rates can be directly attributed to polymorphic differences in ChrY. We have designated this locus as *Y^Cvb3^* (CVB3 susceptibility region of ChrY). With respect to candidates for *Y^Cvb3^*, there are three loci that are biologically relevant to CVB3-induced mortality: *Y^Nkt^* (iNKT-determining region of ChrY) (Wesley *et al.* 2007), *Sry* (sex determining region of ChrY), and *Y^Cmc^* (cardiomyocyte size–determining region of ChrY) (Llamas *et al.* 2007, 2009).

### Differences in basal invariant natural killer T cell numbers do not influence the mortality of CVB3-infected B6-ChrY consomic mice

Invariant natural killer T (iNKT) cells are an important immune regulator during CVB3 infections, and the treatment of mice with α-galactosylceramide, an agonist for iNKT cells, protects the mice from CVB3-induced disease (Liu and Huber 2011; Wu *et al.* 2010). Consequently, *Y^Nkt^*, a locus that leads to a profound deficiency in iNKT cells in male B6-ChrY*^Nkt-129F11/Pas^* mice (Wesley *et al.* 2007), is a functionally relevant candidate for *Y^Cvb3^*. Therefore, we analyzed the number of iNKT cells in the liver, spleen, and thymus of all B6-ChrY consomic strains by staining leukocytes for TCRβ^+^ and CD1d-tetramer^+^ cells and assessing the percentage of iNKT cells by flow cytometry (File S1).

**Figure 3:**
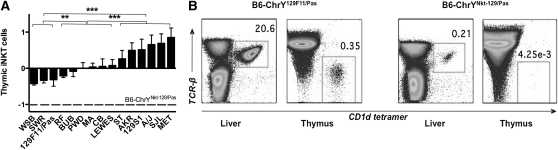
*Y^Nkt^* polymorphism influences the frequency of iNKT cells in B6-ChrY consomic strains. (A) Thymocytes were analyzed by flow cytometry for the percentage of iNKT cells by staining for TCRβ- and CD1d-expressing cells for each B6-ChrY consomic strain and then normalizing to B6 controls. The data are represented as the difference in the percentage iNKT from B6 (B6 is the baseline). The X-axis labels indicate the mouse strain donating ChrY to B6. The hatched line represents the normalized percentage of iNKT cells seen in B6-ChrY^Nkt-129/Pas^ mice. The significance of observed differences among the strains was determined by one-way ANOVA followed by Bonferroni's multiple comparison test. (B) Representative liver and thymus staining from (left) male B6-ChrY^129F11/Pas^ inheriting the ChrY^129F11/Pas^ from mice by natural breeding and (right) male B6-ChrY^Nkt-129/Pas^ mice carrying the ChrY^129/Pas^ transmitted from male founder mice derived from 129/Pas embryonic stem cells. n ≥ 4 mice per group. ^**^P < 0.01; ^***^P < 0.001; ^****^P < 0.0001.

As the various B6-ChrY consomic strains were analyzed throughout multiple experiments, the data derived from each consomic line was normalized to the B6 data from each independent experiment to control for interexperimental variability. Interestingly, we observed a continuous distribution of iNKT cells across the ChrY strains. The analysis of thymic iNKT cells produced three significant groupings (Figure 3A). Similar continuous distributions of iNKT cells were also observed for liver and splenic iNKT cell numbers (data not shown).

**Figure 4:**
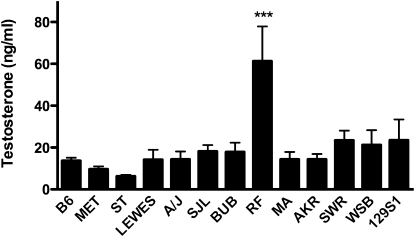
Differences in CVB3-induced mortality are not the result of changes in adult serum testosterone levels among the B6-ChrY consomic strains. Serum testosterone was measured in 8-week-old B6-ChrY consomic male mice and compared with wild-type B6 by EIA. X-axis labels indicate the mouse strain donating ChrY to B6. Only B6-ChrY^RF^ mice exhibited a significant elevation in testosterone compared with B6. Significance of observed differences was determined by one-way ANOVA, followed by a Dunnett’s multiple comparison test. n = 5 mice per strain. ^***^P ≤ 0.001.

Our data confirm that natural ChrY polymorphism results in differences in the percentage of basal iNKT cells in mice. However, it is important to note that further investigation is required to determine whether CVB3 infection itself changes the distribution of iNKT cells among the B6-ChrY consomic strains. Nonetheless, as the strain distribution pattern (SDP) for mortality is discordant with the SDP for iNKT cells in naïve B6-ChrY consomic strains, these data suggest that *Y^Cvb3^* is not *Y^Nkt^*, and that the variations in basal iNKT cell numbers do not contribute to the phenotypic differences in CVB3-induced mortality. Moreover, these data indicate that the *Y^Nkt-129/Pas^* allele transmitted by male founder mice derived from 129F11/Pas embryonic stem cells is unique, as male mice that inherited the wild-type ChrY^129F11/Pas^ by natural breeding differed markedly in iNKT cell numbers (Figure 3B). A direct comparison of these two ChrYs could in theory lead to the identification of the locus controlling iNKT cell numbers and possibly, as with *yaa* (Pisitkun *et al.* 2006; Subramanian *et al.* 2006), the identification of a unique translocation in ChrY^Nkt-129/Pas^ giving rise to a dramatic reduction in iNKT cell numbers compared to naturally occurring *Y^Nkt^* alleles.

### *Sry* allele-dependent variations in prenatal testosterone production do not underlie *Y^Cvb3^*

*Sry* is a particularly intriguing candidate for *Y^Cvb3^* because *Sry* expression by the bipotential cells of the primordial gonad initiates testis differentiation by activating male-specific transcription factors, in particular *Sox9*, to induce Sertoli cell differentiation. This drives testis formation (Kashimada and Koopman 2010), including differentiation of fetal Leydig cells (Brennan *et al.* 2003; Clark *et al.* 2000; Gnessi *et al.* 2000; Yao *et al.* 2002), which produce the androgens required for masculinization of the male fetus during embryogenesis (Sriraman *et al.* 2005). Sertoli cells also contribute to the proper differentiation and function of adult Leydig cells (Loveland and Schlatt 1997; Russell *et al.* 2001; Yan *et al.* 2000) responsible for pubertal production of testosterone and sexual maturation (Ge and Hardy 2007). There is increasing evidence that testis determination may not be the only function of Sry, as it is also expressed in the brain, kidney, and adrenal glands of adult males (Ely *et al.* 2010). The existence of functionally significant *Sry* polymorphisms is well documented in studies using B6-ChrY consomic strains in which *Sry* alleles give rise to varying degrees of sex reversal, ranging from normal testis development to permanent sex reversal (Albrecht *et al.* 2003; Biddle and Nishioka 1988; Eicher and Washburn 1986; Eicher *et al.* 1982; Nagamine *et al.* 1987, 1999). Therefore, *Sry* polymorphisms could lead to differences in neonatal and/or adult testosterone levels, thereby impacting susceptibility to CVB3-induced mortality.

To test our hypothesis, we compared the SDP of *Sry* polymorphisms with the SDP of CVB3-induced mortality. The B6-ChrY consomic lines can be separated into two categories based on the evolutionary origin of the *Mus musculus* species donating ChrY, including *musculus* and *domesticus*. Within the *domesticus* ChrY consomic lines, two groups of mice exist based on their *Sry* alleles (Table 1) (Washburn *et al.* 2001). *Domesticus* Group A is defined as having *Sry* alleles with 13 CAG repeats in glutamine repeat cluster 3, which results in sex reversal when inherited by B6 mice heterozygous for the Chr17 TOrleans mutation. Mice within this group have reduced *Sry* expression in the fetal gonad, which is insufficient for normal development. *Domesticus* Group B is defined as having *Sry* alleles with 12 CAG repeats in glutamine repeat cluster 3 and a 10 bp deletion in the 5′ UTR that allows for normal development in B6 mice heterozygous for the TOrleans mutation and normal *Sry* expression levels in the fetal gonad. However, our analysis of the consomic lines revealed that the SDP for CVB3-induced mortality is discordant with the SDP for *Sry* alleles (Table 1). These data suggest that the *Sry* allelic variations having the potential to influence testis development and neonatal testosterone production are not likely to underlie *Y^Cvb3^*.

**Table 1:** B6-ChrY strains grouped by *Sry* polymorphism

*Musculus* ChrY	*Domesticus* ChrY Group A	*Domesticus* ChrY Group B
B6	*^a^* B6-ChrY^AKR^	*^b^* B6-ChrY^BUB^
*^b^* B6-ChrY^129S1^	*^b^* B6-ChrY^LEWES^	*^b^* B6-ChrY^SJL^
*^a^* B6-ChrY^A/J^	*^a^* B6-ChrY^MA^	*^c^* B6-ChrY^ST^
	*^a^* B6-ChrY^RF^	*^b^* B6-ChrY^SWR^
	*^a^* B6-ChrY^WSB^	
	*^c^* B6-ChrY^MET^	

a Increased susceptibility to CVB3-induced mortality at 100 and 50 PFU compared with B6.

b Increased susceptibility to CVB3-induced mortality at 100 PFU compared with B6.

c Equal susceptibility at both virus doses to CVB3-induced mortality compared with B6.

### Variation in adult testosterone levels do not account for ChrY regulation of CVB3-induced mortality

Testosterone clearly plays a critical role in the sexual dimorphism in susceptibility to myocarditis by regulating both viral infectivity and differences in innate and adaptive antiviral immune responses. Therefore, we hypothesized that allelic differences in *Sry* could also in theory influence the production of postpubertal testosterone in CVB3-infected B6-ChrY consomic mice and lead to differences in mortality among the strains. To test this, blood was collected from male mice at eight weeks of age that were bled between 1 and 3 pm to minimize daily fluctuations in testosterone production. Serum testosterone levels were measured using a testosterone EIA kit (File S1). First, adult testosterone levels did not correlate with *Sry* alleles, suggesting that *Sry* allelic variations with the potential to influence testis development do not influence adult testosterone levels (Table 1). Only B6-ChrY^RF^ consomic mice exhibited significant differences in adult testosterone levels compared with B6 Figure 4. Thus, elevated testosterone levels may contribute to increased mortality in B6-ChrY^RF^, but an increase in adult testosterone levels does not explain the enhanced mortality seen in the other 11 B6-ChrY consomic strains. However, whether ChrY influences other steroid hormones and whether ChrY influences virus receptor expression in the heart independently of testosterone levels remain under consideration.

Our data suggest that *Sry* polymorphisms neither underlie *Y^Cvb3^* nor control adult serum testosterone levels. Nevertheless, dependence of CVB3-induced mortality on both testosterone and ChrY polymorphism makes *Y^Cmc^*, a ChrY polymorphism that impacts the sensitivity of cardiomyocytes to the hypertrophic effects of postpubertal testosterone, a strong candidate for *Y^Cvb3^*. Llamas *et al.* (2007) showed that the surface area of cardiomyocytes from B6 mice is significantly larger than cardiomyocytes from B6-ChrY^A/J^ mice. Orchiectomy of B6 and B6-ChrY^A/J^ consomic mice resulted in a decrease in the size of B6 cardiomyocytes but not in the size of B6-ChrY^A/J^ cardiomyocytes (Llamas *et al.* 2007, 2009). Furthermore, treatment of orchiectomized mice with exogenous testosterone effectively increased the size of cardiomyocytes in B6 mice but not in B6-ChrY^A/J^ mice (Llamas *et al.* 2009). Therefore, these differences are not due to inherent changes in testosterone production between the strains but, rather, due to the insensitivity of cardiomyocytes from B6-ChrY^A/J^ mice to the hypertrophic effects of postpubertal testosterone, which is a direct consequence of *Y^Cmc^*. This same effect may contribute to the differences in CVB3-induced mortality among the B6-ChrY consomic lines, and it is being actively investigated.

The location of *Y^Cvb3^*, as well as *Y^Nkt^* and *Y^Cmc^*, with respect to the pseudoautosomal (PAR) and nonpseudoautosomal (NPAR) regions of ChrY remains to be determined. However, the fact that PAR is free to recombine with the B6 background and that all of the ChrY mice have been backcrossed over 10 generations to B6 excludes *Sts* (steroid sulfatase), the only full-length functional gene within the murine PAR, as a candidate (Park *et al.* 2005). Therefore, *Y^Cvb3^* presumably resides within the NPAR where there are 9 pseudogenes (Gm2098, Gm4017, Gm8498, Gm8502, Gm8506, Gm8510, Gm2303, Gm2316, and Gm2357), 8 validated protein-coding genes of unknown function (Gm2191, Gm6026, Gm16501, Gm4064, Gm10256, Gm10352, Gm3376, and Gm3395), and 13 known genes [*Ddx3y* (DEAD (Asp-Glu-Ala-Asp) box polypeptide 3, Y-linked); *Eif2s3y* (eukaryotic translation initiation factor 2, subunit 3, structural gene Y-linked); *Kdm5d* (lysine (K)-specific demethylase 5D histocompatibility Y); *Rbmy1a1* (RNA binding motif protein, Y chromosome, family 1, member A1); *Sly* (Sycp3 like Y-linked); *Sry* (sex-determining region of ChrY); *Ssty1* (spermiogenesis-specific transcript on the Y 1); *Ssty2* (spermiogenesis-specific transcript on the Y 2); *Ube1y1* (ubiquitin-activating enzyme E1, ChrY 1); *Usp9y* (ubiquitin-specific peptidase 9, Y chromosome); *Uty* (ubiquitously transcribed tetratricopeptide repeat gene, Y chromosome); *Zfy1* (zinc finger protein 1, Y linked); and *Zfy2* (zinc finger protein 2, Y linked)].

Our data suggest that the functional *Sry* alleles influencing testis differentiation are unlikely to be responsible for *Y^Cvb3^* or *Y^Nkt^*. Moreover, a comparison of the available ChrY sequence data (phenome.jax.org) indicates that there are no single nucleotide polymorphisms within the NPAR candidates that uniquely distinguish the *Y^Cvb3^* alleles influencing mortality (Table 1). Thus, it is important to also consider the impact that ChrY structural polymorphism may have on phenotypic differences among the B6-ChrY consomic strains. Structural polymorphism can arise through variations in the number of repeat sequences, inverted sequences, and retroelements composing heterochromatin. As seen in *Drosophila*, Y-linked heterochromatin can influence the epigenetic regulation of autosomal and ChrX gene expression through its interaction with chromatin, thereby epigenetically regulating differences in males (Lemos *et al.* 2008, 2010). Therefore, the differences in CVB3-induced mortality observed among the consomic strains may be the consequence of epigenetic regulation by ChrY resulting from heterochromatic polymorphism.

Delineating the underlying biological components regulating sex differences is critical to appropriate therapeutic treatment of sexually dimorphic diseases. Increasing evidence indicates that ChrY polymorphism can impact biological functions unrelated to male reproduction, such as autoimmune and cardiovascular diseases and hypertension, but the mechanisms behind these alternative functions remain unknown (Llamas *et al.* 2007, 2009; Teuscher *et al.* 2006). Fortunately, the use of ChrY consomic strains of mice presents the opportunity to reveal the polymorphism underlying these unconventional biological functions. Clearly, defining the genetic basis of ChrY functional polymorphism is of considerable importance to human health and disease, particularly in those settings where a male-specific sexual dimorphism dominates.

## Supplementary Material

Supporting Information
