# Supplementary material for: Chromosome Y Regulates Survival Following Murine Coxsackievirus B3 Infection
Source: G3 (Bethesda). 2012 Jan 1;2(1):115–21. doi: 10.1534/g3.111.001610 (PMC3276194; doi:10.1534/g3.111.001610)
Supplement: Supporting Information [file supp_2_1_115__index.html]

Supporting Information 

# Chromosome Y Regulates Survival Following Murine Coxsackievirus B3 Infection

## Supporting Information for Case *et al.*, 2012

**Files in this Data Supplement:**

- File S1 - Supporting Data (.xlsx, 40 KB)
